# A modified three-term PRP conjugate gradient algorithm for optimization models

**DOI:** 10.1186/s13660-017-1373-4

**Published:** 2017-05-03

**Authors:** Yanlin Wu

**Affiliations:** Department of Basic Teaching, Yango College, Fuzhou, Fujian 350015 P.R. China

**Keywords:** 90C26, conjugate gradient, sufficient descent, trust region

## Abstract

The nonlinear conjugate gradient (CG) algorithm is a very effective method for optimization, especially for large-scale problems, because of its low memory requirement and simplicity. Zhang *et al.* (IMA J. Numer. Anal. 26:629-649, 2006) firstly propose a three-term CG algorithm based on the well known Polak-Ribière-Polyak (PRP) formula for unconstrained optimization, where their method has the sufficient descent property without any line search technique. They proved the global convergence of the Armijo line search but this fails for the Wolfe line search technique. Inspired by their method, we will make a further study and give a modified three-term PRP CG algorithm. The presented method possesses the following features: (1) The sufficient descent property also holds without any line search technique; (2) the trust region property of the search direction is automatically satisfied; (3) the steplengh is bounded from below; (4) the global convergence will be established under the Wolfe line search. Numerical results show that the new algorithm is more effective than that of the normal method.

## Introduction

We consider the optimization models defined by

1.1 $$ \min_{x\in\Re^{n}} f(x), $$

where the function $f:\Re^{n}\rightarrow\Re$ is continuously differentiable. There exist many similar professional fields of science that can revert to the above optimization models (see, *e.g.*, [2–21]). The CG method has the following iterative formula for (1.1):

1.2 $$ x_{k+1}=x_{k}+\alpha_{k} d_{k},\quad k=1, 2,\ldots, $$

where $x_{k}$ is the *k*th iterate point, the steplength is $\alpha_{k} > 0$, and the search direction $d_{k}$ is designed by

1.3 $$\begin{aligned} d_{k+1}= \textstyle\begin{cases}-g_{k+1}+\beta_{k}d_{k}, & \mbox{if } k\geq1,\\ -g_{k+1},& \mbox{if }k=0, \end{cases}\displaystyle \end{aligned}$$

where $g_{k}=\nabla f(x_{k})$ is the gradient and $\beta_{k} \in\Re$ is a scalar. At present, there are many well-known CG formulas (see [22–46]) and their applications (see, *e.g.*, [47–50]), where one of the most efficient formulas is the PRP [34, 51] defined by

1.4 $$ \beta_{k}^{\mathrm{PRP}}=\frac{g_{k+1}^{T}\delta_{k}}{ \Vert g_{k} \Vert ^{2}}, $$

where $g_{k+1}=\nabla f(x_{k+1})$ is the gradient, $\delta _{k}=g_{k+1}-g_{k}$, and $\Vert . \Vert $ is the Euclidian norm. The PRP method is very efficient as regards numerical performance, but it fails as regards the global convergence for the general functions under Wolfe line search technique and this is a still open problem; many scholars want to solve it. It is worth noting that a recent work of Yuan *et al.* [52] proved the global convergence of PRP method under a modified Wolfe line search technique for general functions. Al-Baali [53], Gilbert and Nocedal [54], Toouati-Ahmed and Storey [55], and Hu and Storey [56] hinted that the sufficient descent property may be crucial for the global convergence of the conjugate gradient methods including the PRP method. Considering the above suggestions, Zhang, Zhou, and Li [1] firstly gave a three-term PRP formula

1.5 $$\begin{aligned} d_{k+1}= \textstyle\begin{cases} -g_{k+1}+\beta_{k}^{\mathrm{PRP}}d_{k}-\vartheta_{k}\delta_{k}, & \mbox{if } k\geq 1,\\ -g_{k+1},& \mbox{if } k=0, \end{cases}\displaystyle \end{aligned}$$

where $\vartheta_{k}=\frac{g_{k+1}^{T}d_{k}}{ \Vert g_{k} \Vert ^{2}}$. It is not difficult to deduce that $d_{k+1}^{T}g_{k+1}=- \Vert g_{k+1} \Vert ^{2}$ holds for all *k*, which implies that the sufficient descent property is satisfied. Zhang *et al.* proved that the three-term PRP method has global convergence under Armijo line search technique for general functions but this fails for the Wolfe line search. The reason may be the trust region feature of the search direction that cannot be satisfied for this method. In order to overcome this drawback, we will propose a modified three-term PRP formula that will have not only the sufficient descent property but also the trust region feature.

In the next section, a modified three-term PRP formula is given and the new algorithm is stated. The sufficient descent property, the trust region feature, and the global convergence of the new method are established in Section 3. Numerical results are reported in the last section.

## The modified PRP formula and algorithm

Motivated by the above observation, the modified three-term PRP formula is

2.1 $$\begin{aligned} d_{k+1}= \textstyle\begin{cases} -g_{k+1}+\frac{g_{k+1}^{T}\delta_{k}d_{k}-d_{k}^{T}g_{k+1}\delta_{k}}{\gamma _{1} \Vert g_{k} \Vert ^{2}+\gamma_{2} \Vert d_{k} \Vert \delta_{k} \Vert +\gamma_{3} \Vert d_{k} \Vert g_{k} \Vert }, & \mbox{if } k\geq1,\\ -g_{k+1},& \mbox{if } k=0, \end{cases}\displaystyle \end{aligned}$$

where $\gamma_{1}>0$, $\gamma_{2}>0$, and $\gamma_{3}>0$ are constants. It is easy to see that the difference between (1.5) and (2.1) is the denominator of the second and the third terms. This is a little change that will guarantee another good property for (2.1) and impel the global convergence for Wolfe conditions.

### Algorithm 1

New three-term PRP CG algorithm (NTT-PRP-CG-A)

Step 0: Initial given parameters: $x_{1} \in \Re^{n}$, $\gamma_{1}>0$, $\gamma_{2}>0$, $\gamma_{3}>0$, $0<\delta<\sigma<1$, $\varepsilon\in(0,1)$. Let $d_{1}=-g_{1}=-\nabla f(x_{1})$ and $k:=1$.
Step 1: $\Vert g_{k} \Vert \leq\varepsilon$, stop.
Step 2: Get stepsize $\alpha_{k}$ by the following Wolfe line search rules:
  2.2 $$ f(x_{k}+\alpha_{k}d_{k}) \leq f(x_{k})+\delta\alpha_{k} g_{k}^{T}d_{k}, $$
  and
  2.3 $$ g(x_{k}+\alpha_{k}d_{k})^{T}d_{k} \geq\sigma g_{k}^{T}d_{k}. $$
Step 3: Let $x_{k+1}=x_{k}+\alpha_{k}d_{k}$. If the condition $\Vert g_{k+1} \Vert \leq\varepsilon$ holds, stop the program.
Step 4: Calculate the search direction $d_{k+1}$ by (2.1).
Step 5: Set $k:=k+1$ and go to Step 2.

## The sufficient descent property, the trust region feature, and the global convergence

It has been proved that, even for the function $f(x)=\lambda \Vert x \Vert ^{2}$ ($\lambda>0$ is a constant) and the strong Wolfe conditions, the PRP conjugate gradient method may not yield a descent direction for an unsuitable choice (see [24] for details). An interesting feature of the new three-term CG method is that the given search direction is sufficiently descent.

### Lemma 3.1

*The search direction* $d_{k}$ *is defined by* (2.1) *and it satisfies*

3.1 $$ d_{k+1}^{T}g_{k+1} = - \Vert g_{k+1} \Vert ^{2} $$

*and*

3.2 $$ \Vert d_{k+1} \Vert \leq\gamma \Vert g_{k+1} \Vert $$

*for all* $k\geq0$, *where* $\gamma>0$ *is a constant*.

### Proof

For $k=0$, it is easy to get $g_{1}^{T}d_{1}=-g_{1}^{T}g_{1}=- \Vert g_{1} \Vert ^{2}$ and $\Vert d_{1} \Vert = \Vert -g_{1} \Vert = \Vert g_{1} \Vert $, so (3.1) is true and (3.2) holds with $\gamma= 1$.

If $k\geq1$, by (2.1), we have

3.3 $$\begin{aligned} g_{k+1}^{T}d_{k+1} =& - \Vert g_{k+1} \Vert ^{2}+ g_{k+1}^{T}\biggl[ \frac {g_{k+1}^{T}\delta_{k}d_{k}-d_{k}^{T}g_{k+1}\delta_{k}}{\gamma_{1} \Vert g_{k} \Vert ^{2}+\gamma_{2} \Vert d_{k} \Vert \delta_{k} \Vert +\gamma_{3} \Vert d_{k} \Vert g_{k} \Vert }\biggr] \\ =& - \Vert g_{k+1} \Vert ^{2}+\frac{g_{k+1}^{T}\delta_{k} g_{k+1}^{T}d_{k}-d_{k}^{T}g_{k+1}g_{k+1}^{T} \delta_{k}}{\gamma_{1} \Vert g_{k} \Vert ^{2}+\gamma_{2} \Vert d_{k} \Vert \delta_{k} \Vert +\gamma_{3} \Vert d_{k} \Vert g_{k} \Vert } \\ =&- \Vert g_{k+1} \Vert ^{2}. \end{aligned}$$

Then (3.1) is satisfied. By (2.1) again, we obtain

3.4 $$ \begin{aligned}[b] \Vert d_{k+1} \Vert &= \biggl\Vert g_{k+1}+\frac{g_{k+1}^{T}\delta _{k}d_{k}-d_{k}^{T}g_{k+1}\delta_{k}}{\gamma_{1} \Vert g_{k}\Vert^{2}+\gamma_{2} \Vert d_{k}\Vert\delta_{k} \Vert+\gamma_{3}\Vert d_{k} \Vert g_{k}\Vert} \biggr\Vert \\ &\leq \Vert g_{k+1} \Vert+\frac{\Vert g_{k+1}^{T}\delta _{k}d_{k}-d_{k}^{T}g_{k+1} \delta_{k} \Vert}{\gamma_{1}\Vert g_{k} \Vert ^{2}+\gamma_{2}\Vert d_{k} \Vert\delta_{k}\Vert+\gamma_{3} \Vert d_{k}\Vert g_{k} \Vert} \\ &\leq\Vert g_{k+1} \Vert+\frac{\Vert\delta_{k} \Vert \Vert g_{k+1} \Vert\Vert d_{k} \Vert+\Vert d_{k} \Vert\Vert g_{k+1} \Vert\Vert \delta_{k} \Vert}{\gamma_{1}\Vert g_{k} \Vert^{2}+\gamma_{2}\Vert d_{k} \Vert \delta_{k}\Vert+\gamma_{3} \Vert d_{k}\Vert g_{k} \Vert} \\ &\leq\Vert g_{k+1} \Vert+\frac{2\Vert\delta_{k} \Vert \Vert g_{k+1} \Vert\Vert d_{k} \Vert}{\gamma_{2}\Vert d_{k} \Vert\delta_{k}\Vert } \\ &= (1+2/\gamma_{2})\Vert g_{k+1} \Vert, \end{aligned} $$

where the last inequality follows from $\frac{1}{\gamma_{1} \Vert g_{k} \Vert ^{2}+\gamma_{2} \Vert d_{k} \Vert \delta_{k} \Vert +\gamma_{3} \Vert d_{k} \Vert g_{k} \Vert }\leq\frac {1}{\gamma_{2} \Vert d_{k} \Vert \delta_{k}\Vert}$. Thus (3.2) holds for all $k\geq0$ with $\gamma=\max\{1,1+2/\gamma_{2}\}$. The proof is complete. □

### Remark

(1) Equation (3.1) is the sufficient descent property and the inequality (3.2) is the trust region feature. And these two relations are verifiable without any other conditions.

(2) Equations (3.1) and (2.2) imply that the sequence $\{ f(x_{k})\}$ generated by Algorithm 1 is descent, namely $f(x_{k}+\alpha _{k}d_{k})\leq f(x_{k})$ holds for all *k*.

To establish the global convergence of Algorithm 1, the normal conditions are needed.

### Assumption A

(i) The defined level set $\Omega=\{x\in\Re^{n}\mid f(x)\leq f(x_{1})\}$ is bounded with given point $x_{1}$.
(ii) The function *f* has a lower bound and it is differentiable. The gradient *g* is Lipschitz continuous
  3.5 $$ \bigl\Vert g(x)-g(y) \bigr\Vert \leq L \Vert x-y \Vert , \quad \forall x,y\in\Re^{n}, $$
  where $L>0$ a constant.

### Lemma 3.2

*Suppose that Assumption* A *holds and NTT*-*PRP*-*CG*-*A generates the sequence* $\{x_{k},d_{k},\alpha_{k},g_{k}\}$. *Then there exists a constant* $\beta >0$ *such that*

3.6 $$ \alpha_{k}\geq\beta,\quad\forall k\geq1. $$

### Proof

Using (3.5) and (2.3) generate

$$\begin{aligned} \alpha_{k}L \geq& (g_{k+1}-g_{k})^{T}d_{k} \\ \geq& -(1-\sigma)g_{k}^{T}d_{k} \\ =& (1-\sigma) \Vert g_{k} \Vert ^{2}, \end{aligned}$$

where the last equality follows from (3.1). By (3.2), we get

$$\alpha_{k}\geq\frac{1-\sigma}{L}\frac{ \Vert g_{k} \Vert ^{2}}{ \Vert d_{k} \Vert ^{2}}\geq \frac{1-\sigma}{L\gamma}. $$

Setting $\beta\in(0,\frac{1-\sigma}{L\gamma})$ completes the proof. □

### Remark

The above lemma shows that the steplengh $\alpha_{k}$ has a lower bound, which is helpful for the global convergence of Algorithm 1.

### Theorem 3.1

*Let the conditions of Lemma* 3.2 *hold and* $\{x_{k},d_{k},\alpha_{k},g_{k}\}$ *be generated by NTT*-*PRP*-*CG*-*A*. *Thus we get*

$$\lim_{k\rightarrow\infty} \Vert g_{k} \Vert =0. $$

### Proof

By (2.2), (3.1), and (3.6), we have

$$\delta\beta \Vert g_{k} \Vert ^{2} \leq\delta \alpha_{k} \Vert g_{k} \Vert ^{2} \leq f(x_{k})-f(x_{k}+\alpha_{k}d_{k}). $$

Summing the above inequality from $k=1$ to ∞, we have

$$\sum_{k=1}^{\infty}\delta\beta \Vert g_{k} \Vert ^{2} \leq f(x_{1})-f_{\infty}\leq\infty, $$

which means that

$$\Vert g_{k} \Vert \rightarrow0,\quad k\rightarrow\infty. $$

The proof is complete. □

## Numerical results and discussion

This section will report the numerical experiment of the NTT-PRP-CG-A and the algorithm of Zhang *et al.* [1] (called Norm-PRP-A), where the Norm-PRP-A is the Step 4 of Algorithm 1 that is replaced by: Calculate the search direction $d_{k+1}$ by (1.5). Since the method is based on the search direction (1.5), we only compare the numerical results between the new algorithm and the Norm-PRP-A. The codes of these two algorithms are written by Matlab and the problems are chosen from [57, 58] with given initial points. The tested problems are listed in Table 1. The parameters are $\gamma_{1}=2$, $\gamma_{2}=5$, $\gamma_{3}=3$, $\delta=0.01$, $\sigma=0.86$. The program uses the *Himmelblau* rule: Set $St_{1}=\frac{ \vert f(x_{k})-f(x_{k+1}) \vert }{ \vert f(x_{k}) \vert }$ if $\vert f(x_{k}) \vert > \tau_{1}$, otherwise set $St_{1}= \vert f(x_{k})-f(x_{k+1}) \vert $. The program stops if $\Vert g(x) \Vert <\epsilon$ or $St_{1} < \tau_{2}$ hold, where we choose $\epsilon=10^{-6}$ and $\tau_{1}=\tau _{2}=10^{-5}$. For the choice of the stepsize $\alpha_{k}$ in (2.2) and (2.3), the largest cycle number is 10 and the current stepsize is accepted. The dimensions of the test problems accord to large-scale variables with 3,000, 12,000, and 30,000. The runtime environment is MATLAB R2010b and run on a PC with CPU Intel Pentium(R) Dual-Core CPU at 3.20 GHz, 2.00 GB of RAM, and the Windows 7 operating system.

**Table 1:** **Test problems**

**No.**	**Problems**	$\boldsymbol{x_{0}}$
1	Extended Freudenstein and Roth function	[0.5,−2,…,0.5,−2]
2	Extended trigonometric function	[0.2,0.2,…,0.2]
3	Extended Rosenbrock function	[−1.2,1,−1.2,1,…,−1.2,1]
4	Extended White and Holst function	[−1.2,1,−1.2,1,…,−1.2,1]
5	Extended Beale function	[1,0.8,…,1,0.8]
6	Extended penalty function	[1,2,3,…,*n*]
7	Perturbed quadratic function	[0.5,0.5,…,0.5]
8	Raydan 1 function	[1,1,…,1]
9	Raydan 2 function	[1,1,…,1]
10	Diagonal 1 function	[1/*n*,1/*n*,…,1/*n*]
11	Diagonal 2 function	[1/1,1/2,…,1/*n*]
12	Diagonal 3 function	[1,1,…,1]
13	Hager function	[1,1,…,1]
14	Generalized tridiagonal 1 function	[2,2,…,2]
15	Extended tridiagonal 1 function	[2,2,…,2]
16	Extended three exponential terms function	[0.1,0.1,…,0.1]
17	Generalized tridiagonal 2 function	[−1,−1,…,−1,−1]
18	Diagonal 4 function	[1,1,…,1,1]
19	Diagonal 5 function	[1.1,1.1,…,1.1]
20	Extended Himmelblau function	[1,1,…,1]
21	Generalized PSC1 function	[3,0.1,…,3,0.1]
22	Extended PSC1 function	[3,0.1,…,3,0.1]
23	Extended Powell function	[3,−1,0,1,…]
24	Extended block diagonal BD1 function	[0.1,0.1,…,0.1]
25	Extended Maratos function	[1.1,0.1,…,1.1,0.1]
26	Extended Cliff function	[0,−1,…,0,−1]
27	Quadratic diagonal perturbed function	[0.5,0.5,…,0.5]
28	Extended Wood function	[−3,−1,−3,−1,…,−3,−1]
29	Extended Hiebert function	[0,0,…,0]
30	Quadratic QF1 function	[1,1,…,1]
31	Extended quadratic penalty QP1 function	[1,1,…,1]
32	Extended quadratic penalty QP2 function	[1,1,…,1]
33	Quadratic QF2 function	[0.5,0.5,…,0.5]
34	Extended EP1 function	[1.5.,1.5.,…,1.5]
35	Extended tridiagonal-2 function	[1,1,…,1]
36	BDQRTIC function (CUTE)	[1,1,…,1]
37	TRIDIA function (CUTE)	[1,1,…,1]
38	ARWHEAD function (CUTE)	[1,1,…,1]
39	NONDIA (Shanno-78) function (CUTE)	[−1,−1,…,−1]
40	NONDQUAR function (CUTE)	[1,−1,1,−1,…,1,−1]
41	DQDRTIC function (CUTEr)	[3,3,3...,3]
42	EG2 function (CUTE)	[1,1,1...,1]
43	DIXMAANA function (CUTE)	[2,2,2,…,2]
44	DIXMAANB function (CUTE)	[2,2,2,…,2]
45	DIXMAANC function (CUTE)	[2,2,2,…,2]
46	DIXMAANE function (CUTE)	[2,2,2,…,2]
47	Partial perturbed quadratic function	[0.5,0.5,…,0.5]
48	Broyden tridiagonal function	[−1,−1,…,−1]
49	Almost perturbed quadratic function	[0.5,0.5,…,0.5]
50	Tridiagonal perturbed quadratic function	[0.5,0.5,…,0.5]
51	EDENSCH function (CUTE)	[0,0,…,0]
52	VARDIM function (CUTE)	[1 − 1/*n*,1 − 2/*n*,…,1 − *n*/*n*]
53	STAIRCASE S1 function	[1,1,…,1]
54	LIARWHD function (CUTEr)	[4,4,…,4]
55	DIAGONAL 6 function	[1,1,…,1]
56	DIXON3DQ function (CUTE)	[−1,−1,…,−1]
57	DIXMAANF function (CUTE)	[2,2,2,…,2]
58	DIXMAANG function (CUTE)	[2,2,2,…,2]
59	DIXMAANH function (CUTE)	[2,2,2,…,2]
60	DIXMAANI function (CUTE)	[2,2,2,…,2]
61	DIXMAANJ function (CUTE)	[2,2,2,…,2]
62	DIXMAANK function (CUTE)	[2,2,2,…,2]
63	DIXMAANL function (CUTE)	[2,2,2,…,2]
64	DIXMAAND function (CUTE)	[2,2,2,…,2]
65	ENGVAL1 function (CUTE)	[2,2,2,…,2]
66	FLETCHCR function (CUTE)	[0,0,…,0]
67	COSINE function (CUTE)	[1,1,…,1]
68	Extended DENSCHNB function (CUTE)	[1,1,…,1]
69	DENSCHNF function (CUTEr)	[2,0,2,0,…,2,0]
70	SINQUAD function (CUTE)	[0.1,0.1,…,0.1]
71	BIGGSB1 function (CUTE)	[0,0,…,0]
72	Partial perturbed quadratic PPQ2 function	[0.5,0.5,…,0.5]
73	Scaled quadratic SQ1 function	[1,2,…,*n*]
74	Scaled quadratic SQ2 function	[1,2,…,*n*]

Table 2 report the test numerical results of the NTT-PRP-CG-A and the Norm-PRP-A, and we notate:

**Table 2:** **Numerical results**

**No.**	**Dimension**	**NTT-PRP-CG-A**			**Norm-PRP-A**		
		**Ni**	**Nfg**	**CPU time**	**Ni**	**Nfg**	**CPU time**
1	3,000	15	43	0.468003	31	92	0.546004
	12,000	15	43	0.842405	56	158	1.778411
	30,000	15	43	1.482009	36	113	2.730018
2	3,000	57	131	0.374402	55	126	0.374402
	12,000	63	144	1.138807	62	142	0.920406
	30,000	66	152	3.08882	66	152	2.511616
3	3,000	54	186	0.124801	117	375	0.202801
	12,000	67	233	0.234001	144	479	0.514803
	30,000	73	238	0.530403	159	522	1.62241
4	3,000	59	198	0.296402	207	595	0.936006
	12,000	34	139	0.733205	264	801	4.305628
	30,000	74	256	4.118426	228	618	8.907657
5	3,000	23	68	0.093601	39	106	0.124801
	12,000	23	69	0.265202	39	109	0.390003
	30,000	21	64	0.826805	47	135	1.279208
6	3,000	80	185	0.124801	80	185	0.093601
	12,000	103	232	0.405603	103	232	0.343202
	30,000	102	235	1.216808	102	235	0.998406
7	3,000	1,000	2,002	1.045207	357	943	0.421203
	12,000	1,000	2,002	3.16682	835	2,257	2.808018
	30,000	1,000	2,002	9.781263	1,000	2,779	9.734462
8	3,000	21	47	0.0468	19	46	0.0312
	12,000	20	44	0.093601	19	46	0.093601
	30,000	20	44	0.296402	19	46	0.265202
9	3,000	12	26	0.0312	12	26	0.0312
	12,000	12	26	0.0468	12	26	0.0624
	30,000	12	26	0.202801	12	26	0.156001
10	3,000	2	13	0.0312	2	13	0.0312
	12,000	2	13	0.124801	2	13	0.093601
	30,000	2	13	0.312002	2	13	0.280802
11	3,000	81	194	0.171601	24	101	0.0624
	12,000	91	247	0.764405	15	59	0.202801
	30,000	11	35	0.436803	13	50	0.280802
12	3,000	17	36	0.0468	14	33	0.0624
	12,000	19	40	0.171601	14	33	0.124801
	30,000	19	40	0.499203	14	33	0.343202
13	3,000	23	86	0.093601	22	84	0.078
	12,000	42	111	0.452403	42	111	0.468003
	30,000	2	13	0.358802	2	13	0.327602
14	3,000	6	15	0.717605	6	15	0.733205
	12,000	6	15	7.004445	5	13	5.709637
	30,000	3	8	14.258491	3	8	13.587687
15	3,000	38	85	1.794011	66	176	3.04202
	12,000	41	102	17.924515	60	169	28.09578
	30,000	44	114	75.395283	68	194	120.245571
16	3,000	20	42	0.0624	20	42	0
	12,000	24	50	0.171601	24	50	0.156001
	30,000	24	50	0.483603	24	50	0.436803
17	3,000	24	55	0.156001	31	71	0.218401
	12,000	33	73	0.764405	29	74	0.717605
	30,000	48	103	3.042019	30	81	1.996813
18	3,000	3	10	0.0156	13	43	0.0312
	12,000	3	10	0.0312	13	43	0.0156
	30,000	3	10	0.0312	14	47	0.124801
19	3,000	3	9	0	3	9	0
	12,000	3	9	0.0468	3	9	0.0312
	30,000	3	9	0.124801	3	9	0.124801
20	3,000	33	82	0.0312	26	74	0.0312
	12,000	11	61	0.0624	5	35	0.0312
	30,000	5	35	0.093601	20	67	0.218401
21	3,000	25	59	0.093601	27	63	0.0624
	12,000	27	63	0.249602	26	60	0.187201
	30,000	25	58	0.530403	27	63	0.530403
22	3,000	6	31	0.0312	7	42	0
	12,000	6	31	0.0624	5	21	0.0624
	30,000	6	31	0.218401	5	21	0.124801
23	3,000	134	383	0.670804	334	986	1.52881
	12,000	147	416	2.652017	452	1,309	7.73765
	30,000	114	330	5.304034	291	854	12.776482
24	3,000	28	90	0.0624	50	126	0.109201
	12,000	31	108	0.249602	60	146	0.405603
	30,000	28	97	0.686404	67	160	1.170007
25	3,000	28	56	0.0312	28	56	0.0312
	12,000	7	16	0.0156	231	774	0.748805
	30,000	7	16	0.0312	213	774	2.028013
26	3,000	65	152	0.124801	65	152	0.124801
	12,000	72	166	0.514803	72	166	0.468003
	30,000	79	180	1.51321	79	180	1.341609
27	3,000	31	94	0.0624	104	327	0.156001
	12,000	43	137	0.187201	202	655	0.639604
	30,000	104	329	1.154407	384	1,231	4.024826
28	3,000	40	124	0.0468	31	76	0.0312
	12,000	31	91	0.124801	38	95	0.124801
	30,000	40	107	0.546003	32	78	0.265202
29	3,000	4	19	0.0312	100	287	0.124801
	12,000	4	19	0.0156	84	240	0.312002
	30,000	4	19	0.093601	93	264	0.842405
30	3,000	1,000	2,002	0.842405	446	1,205	0.436803
	12,000	1,000	2,002	2.636417	754	2,010	2.074813
	30,000	1,000	2,002	8.330453	1,000	2,721	8.065252
31	3,000	29	66	0.0468	29	66	0.0624
	12,000	34	78	0.156001	34	78	0.156001
	30,000	34	78	0.421203	34	78	0.452403
32	3,000	48	100	0.093601	48	100	0.093601
	12,000	37	80	0.280802	37	80	0.234001
	30,000	36	80	0.780005	36	80	0.670804
33	3,000	3	7	0	3	7	0
	12,000	2	5	0	2	5	0.0312
	30,000	2	5	0.0312	2	5	0
34	3,000	4	8	0.0312	4	8	0.0312
	12,000	7	14	0.0624	7	14	0.0312
	30,000	10	20	0.156001	10	20	0.124801
35	3,000	12	24	0.0312	12	24	0
	12,000	21	42	0.093601	21	42	0.093601
	30,000	4	10	0.093601	4	10	0.0312
36	3,000	14	48	1.138807	45	148	3.244821
	12,000	8	28	6.910844	120	369	95.831414
	30,000	17	55	55.427155	162	483	488.922734
37	3,000	776	1,559	0.733205	1,000	2,688	1.107607
	12,000	1,000	2,006	3.322821	1,000	2,733	3.556823
	30,000	1,000	2,011	9.828063	506	1,378	4.960832
38	3,000	9	30	0.0312	27	81	0.0312
	12,000	10	32	0.0468	21	60	0.140401
	30,000	11	34	0.140401	24	69	0.312002
39	3,000	26	52	0.0624	26	52	0
	12,000	29	58	0.093601	29	58	0.093601
	30,000	23	46	0.187201	23	46	0.171601
40	3,000	554	1,332	5.881238	1,000	2,856	11.013671
	12,000	1,000	2,228	39.733455	1,000	2,892	43.352678
	30,000	1,000	2,247	100.745446	1,000	2,866	108.186694
41	3,000	27	68	0.078	49	133	0.0312
	12,000	28	69	0.093601	50	136	0.124801
	30,000	37	91	0.390002	39	101	0.374402
42	3,000	6	24	0.0312	6	24	0
	12,000	6	24	0.0624	6	24	0.0624
	30,000	6	24	0.187201	6	24	0.156001
43	3,000	28	60	0.202801	28	60	0.218401
	12,000	30	64	0.936006	30	64	0.858005
	30,000	32	68	2.527216	31	66	2.230814
44	3,000	46	96	0.358802	46	96	0.296402
	12,000	49	102	1.51321	49	102	1.404009
	30,000	52	108	4.024826	52	108	3.728424
45	3,000	19	44	0.202801	19	44	0.124801
	12,000	20	46	0.608404	20	46	0.577204
	30,000	20	46	1.54441	20	46	1.48201
46	3,000	117	244	0.920406	108	296	0.967206
	12,000	165	340	5.116833	120	326	4.009226
	30,000	195	400	15.678101	126	341	10.576868
47	3,000	27	66	8.299253	44	102	12.963683
	12,000	31	87	93.741001	49	141	150.150963
	30,000	69	182	1,163.84546	85	256	1,490.683156
48	3,000	32	74	1.762811	27	63	1.310408
	12,000	50	103	30.154993	29	74	19.546925
	30,000	42	100	112.726323	37	87	94.209004
49	3,000	1,000	2,002	0.858005	575	1,593	0.577204
	12,000	1,000	2,002	2.792418	885	2,377	2.527216
	30,000	1,000	2,002	9.484861	1,000	2,738	8.143252
50	3,000	1,000	2,002	57.236767	370	998	23.727752
	12,000	1,000	2,002	617.73276	920	2,495	676.233135
	30,000	1,000	2,002	2,467.96702	1,000	2,720	2,856.471911
51	3,000	23	48	0.124801	23	48	0.140401
	12,000	23	48	0.811205	23	48	0.452403
	30,000	23	48	1.154407	23	48	1.216808
52	3,000	121	276	0.436803	121	276	0.374402
	12,000	138	316	2.090413	138	316	1.684811
	30,000	150	344	4.66443	150	344	4.61763
53	3,000	1,000	2,009	0.998406	1,000	2,706	1.170008
	12,000	1,000	2,009	3.759624	1,000	2,661	3.369622
	30,000	1,000	2,009	8.502054	1,000	2,781	9.594061
54	3,000	32	87	0.0624	203	577	0.343202
	12,000	13	41	0.109201	201	607	1.029607
	30,000	42	99	0.483603	362	1,112	4.836031
55	3,000	21	44	0.546004	21	44	0.530403
	12,000	23	48	7.488048	23	48	7.441248
	30,000	24	50	30.654197	24	50	29.983392
56	3,000	430	886	0.358802	507	1,397	0.608404
	12,000	430	886	1.450809	613	1,667	2.043613
	30,000	430	886	3.541223	491	1,337	4.492829
57	3,000	145	296	1.154407	55	132	0.468003
	12,000	207	420	7.75325	69	179	2.246414
	30,000	265	536	19.500125	77	196	6.27124
58	3,000	107	223	0.873606	81	202	0.670804
	12,000	124	257	3.931225	91	243	3.07322
	30,000	142	293	10.514467	98	261	8.205653
59	3,000	77	166	0.639604	52	137	0.405603
	12,000	107	226	4.508429	60	152	1.934412
	30,000	94	203	7.082445	72	181	5.803237
60	3,000	488	983	3.978026	111	303	0.967206
	12,000	175	360	5.522435	106	293	3.650423
	30,000	194	398	14.476893	140	377	11.856076
61	3,000	145	296	1.185608	56	142	0.468003
	12,000	206	418	6.692443	70	179	2.277615
	30,000	264	534	19.390924	92	247	7.75325
62	3,000	153	314	1.232408	63	163	0.717605
	12,000	239	486	7.332047	86	214	2.761218
	30,000	313	634	23.166148	96	261	8.127652
63	3,000	209	430	1.934412	138	378	1.388409
	12,000	1,000	2,009	37.799042	164	448	6.489642
	30,000	1,000	2,009	87.220159	191	521	18.532919
64	3,000	29	64	0.265202	29	64	0.218401
	12,000	31	68	1.045207	31	68	0.936006
	30,000	32	70	2.340015	32	70	2.324415
65	3,000	22	51	1.903212	19	45	1.59121
	12,000	17	38	14.586094	17	38	14.258491
	30,000	17	38	61.167992	17	38	59.420781
66	3,000	1,000	2,003	57.985572	733	2,293	50.684725
	12,000	1,000	2,003	618.637566	214	671	171.757101
	30,000	4	11	10.374067	58	157	163.879051
67	3,000	6	37	0.0312	9	59	0.0312
	12,000	10	63	0.499203	48	231	0.577204
	30,000	5	27	0.124801	10	54	0.296402
68	3,000	35	72	0.0312	35	72	0.0312
	12,000	38	78	0.124801	38	78	0.109201
	30,000	39	80	0.343202	39	80	0.374402
69	3,000	27	58	0.0312	30	64	0.0624
	12,000	28	60	0.140401	32	68	0.187201
	30,000	29	62	0.421203	33	70	0.468003
70	3,000	25	82	1.950013	129	386	8.876457
	12,000	52	184	46.8471	143	479	119.621567
	30,000	13	62	52.790738	193	598	597.967433
71	3,000	1,000	2,004	0.889206	449	1,247	0.468003
	12,000	1,000	2,004	4.196427	661	1,779	2.106014
	30,000	1,000	2,004	7.238446	606	1,645	5.506835
72	3,000	706	2,011	228.837867	1,000	2,845	323.405673
	12,000	569	1,589	1,742.46877	785	2,234	2,412.040662
	30,000	229	654	3,931.381201	1,000	2,813	17,084.27791
73	3,000	1,000	2,002	0.936006	490	1,307	0.421203
	12,000	1,000	2,002	3.291621	900	2,460	2.605217
	30,000	1,000	2,002	7.566048	1,000	2,735	7.940451
74	3,000	1,000	2,002	0.873606	398	1,061	0.374402
	12,000	1,000	2,002	4.399228	795	2,120	2.262015
	30,000	1,000	2,002	7.519248	1,000	2,682	7.86245

No. the test problems number. Dimension: the variables number.

Ni: the iteration number. Nfg: the function and the gradient value number. CPU time: the CPU time of operating system in seconds.

**Figure 1:**
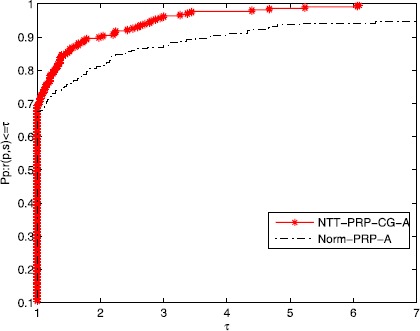
**Performance profiles of the algorithms for the test problems (Ni).**

**Figure 2:**
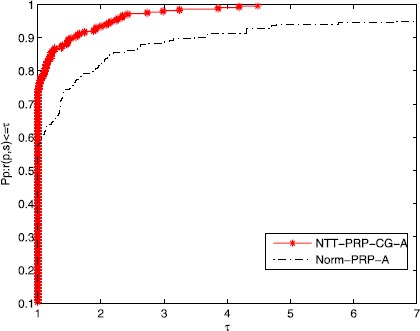
**Performance profiles of the algorithms for the test problems (Nfg).**

**Figure 3:**
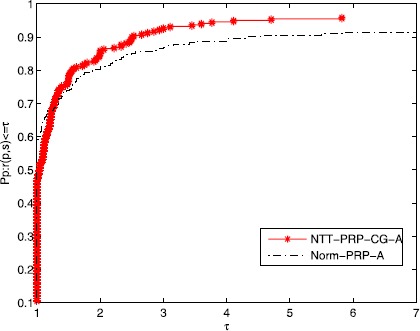
**Performance profiles of the algorithms for the test problems (CPU time).**

A new tool was given by Dolan and Moré [59] to analyze the performance of the algorithms. Figures 1-3 show that the efficiency of the NTT-PRP-CG-A and the Norm-PRP-A relate to Ni, Nfg, and CPU time, respectively. It is easy to see that these two algorithms are effective for those problems and the given three-term PRP conjugate gradient method is more effective than that of the normal three-term PRP conjugate gradient method. Moreover, the NTT-PRP-CG-A has good robustness. Overall, the presented algorithm has some potential property both in theory and numerical experiment, which is noticeable.

## Conclusions

In this paper, based on the PRP formula for unconstrained optimization, a modified three-term PRP CG algorithm was presented. The proposed method possesses sufficient descent property also holds without any line search technique, and we have automatically the trust region property of the search direction. Under the Wolfe line search, the global convergence was proven. Numerical results showed that the new algorithm is more effective compared with the normal method.
